# Survey and online discussion groups to develop a patient-rated outcome measure on acceptability of treatment response in vitiligo

**DOI:** 10.1186/1471-5945-14-10

**Published:** 2014-06-14

**Authors:** Selina K Tour, Kim S Thomas, Dawn-Marie Walker, Paul Leighton, Adrian SW Yong, Jonathan M Batchelor

**Affiliations:** 1 Centre of Evidence Based Dermatology, The University of Nottingham, Nottingham, UK; 2 Faculty of Medicine and Health Sciences, University of Nottingham, Nottingham, UK; 3 Norfolk and Norwich University Hospitals NHS Foundation Trust, Norwich, UK

**Keywords:** Vitiligo, Outcome measure, Patient-reported outcome, Randomised controlled trial

## Abstract

**Background:**

Vitiligo is a chronic depigmenting skin disorder which affects around 0.5-1% of the world’s population. The outcome measures used most commonly in trials to judge treatment success focus on repigmentation. Patient-reported outcome measures of treatment success are rarely used, although recommendations have been made for their inclusion in vitiligo trials. This study aimed to evaluate the face validity of a new patient-reported outcome measure of treatment response, for use in future trials and clinical practice.

**Method:**

An online survey to gather initial views on what constitutes treatment success for people with vitiligo or their parents/carers, followed by online discussion groups with patients to reach consensus on what constitutes treatment success for individuals with vitiligo, and how this can be assessed in the context of trials. Participants were recruited from an existing database of vitiligo patients and through posts on the social network sites Facebook and Twitter.

**Results:**

A total of 202 survey responses were received, of which 37 were excluded and 165 analysed. Three main themes emerged as important in assessing treatment response: a) the match between vitiligo and normal skin (how well it blends in); b) how noticeable the vitiligo is and c) a reduction in the size of the white patches. The majority of respondents said they would consider 80% or more repigmentation to be a worthwhile treatment response after 9 months of treatment. Three online discussion groups involving 12 participants led to consensus that treatment success is best measured by asking patients how noticeable their vitiligo is after treatment. This was judged to be best answered using a 5-point Likert scale, on which a score of 4 or 5 represents treatment success.

**Conclusions:**

This study represents the first step in developing a patient reported measure of treatment success in vitiligo trials. Further work is now needed to assess its construct validity and responsiveness to change.

## Background

Vitiligo is a chronic depigmenting skin disease characterised by loss of skin colour in patches [1]. It affects people of all ages, ethnic groups and skin types [2,3] and around 0.5%-1% of the world’s population [1,2] although estimates are higher in countries and cultures where the stigma of the skin disease may be higher [1].

There is no cure for vitiligo but there are numerous treatment options. These include topical and oral preparations, light therapy, surgical procedures, psychological and complementary therapies [1]. The only licensed treatment for vitiligo in the UK is cosmetic camouflage [3], although many other treatments are used in clinical practice.

Physical symptoms in vitiligo are usually mild, but the unpredictable nature of the disease and its tendency to progress in the majority of cases can be psychologically and cosmetically overwhelming [1,2,4]. Living with vitiligo can be a continuous struggle, with the psychological characteristics of each individual determining their ability to adjust to and cope with disfigurement [5].

Although clinical studies have assessed many treatments for vitiligo, the heterogeneity of these studies makes comparison of the effectiveness of treatments – alone or combined – very difficult [1,2,6]. The updated Cochrane systematic review of interventions for vitiligo published in 2010 [1] and other reviews have highlighted problems such as variance in design and a lack of standardised outcome measures and scales used in clinical trials [1,2,6,7]. There is a pressing need to develop core outcome measures, so that effectiveness of treatments can be compared and combined more easily across trials 2].

It is important that outcomes used in trials are relevant to patients as well as clinicians [2]. Repigmentation is the most frequently used outcome measure and is typically captured using either clinical assessments or through digital images [2]. Vitiligo results in patches of depigmented skin, so intuitively it would seem to be a simple matter of recording treatment success or failure based on changes in the amount of repigmented skin. However, repigmentation of vitiligo can often be uneven, resulting in a poor cosmetic result from a patient’s perspective.

Despite recommendations for the inclusion of patients’ views when evaluating interventions [1,6], patient-reported outcomes have not been commonly used in trial to date [2], and validated tools are lacking.

The aim of the study was to develop and provide preliminary data on the face validity of a patient-reported outcome measure of the acceptability of treatment response.

Specific objectives were:

•To conduct patient surveys and online discussion groups to establish the most appropriate form of wording and scale to use.

•To ensure that the wording of the question assessing satisfaction with treatment response is relevant to, and easily understood by, vitiligo patients (face validity).

•To establish what represents clinically worthwhile treatment response from a patient’s perspective.

In addition to improving our understanding of what constitutes a successful treatment response from a patient perspective, it is anticipated that the resulting outcome measure will be useful for use in future vitiligo trials and clinical practice. The results will also be used to inform an ongoing international initiative to establish a core outcome set for use in vitiligo trials [8].

## Methods

This project was conducted in two stages. First, an online survey was used to identify which aspects of treatment response are most important to patients.

The online survey was followed by three separate online discussion groups, in which the results of the survey were explored with patients, and consensus was reached regarding the most appropriate form of wording for the proposed patient-reported outcome measure.

The project was approved by the University of Nottingham’s Medical School Research Ethics committee (Ethics Reference No. LTg15082013 SoM Dermatol).

### Online survey

#### Participants

Participants were recruited from an existing mailing list held at the Centre of Evidence-Based Dermatology at the University of Nottingham. This list consisted of individuals who had participated in a previous Vitiligo Priority Setting Partnership [7] and those who had contacted us expressing interest in being involved in vitiligo research. In addition, participants were recruited through the UK Vitiligo Society Facebook Page, and details of the survey were ‘tweeted’ under the UK Dermatology Clinical Trials Network Twitter feed. Participants in the survey included both those who had sought treatment for their vitiligo and those who had not, and included parents/guardians of children with vitiligo as well as those with vitiligo themselves. We did not include clinicians and healthcare professionals who had participated in the previous Priority Setting Partnership. Efforts were made to ensure broad representation across all age and ethnic groups. Although recruitment was targeted largely at participants in the UK, there were no exclusions based on country of residence and details of nationality were recorded.

#### Survey distribution

The survey, which took approximately 5minutes to complete was created using Survey Monkey software [9] and consisted of 14 questions. No incentives were offered for participation. Prior to distribution, we piloted it by asking a group of clinicians, researchers and members of the Centre of Evidence-Based Dermatology (CEBD) Patient Panel to review the survey and comment on the relevance of the survey content and how easy it was to understand and complete.

Details of the survey and information sheets were emailed to 188 potential participants from an existing mailing list held at the CEBD that included patients who had previously expressed an interest in finding out more about vitiligo research. The survey was open from 29^th^ July 2013 until the 19^th^ August 2013. Two reminders were emailed to all on the mailing list, and additional posts were placed on the Vitiligo Society’s Facebook page and the UK Dermatology Clinical Trials Network Twitter feed in order to broaden recruitment. Completion of more than 1 survey question implied consent to participate.

Data collection included demographic details; the extent of the vitiligo and previous treatments used; opinions on what a ‘cosmetically acceptable response’ to treatment meant to the participant and whether they felt it was the same as ‘satisfaction with the result’. A selection of 11 words and phrases to describe treatment response were also presented, from which participants chose the most meaningful to them (see Results section).

Participants were also asked to look at a series of images featuring a young boy with dark skin with a vitiliginous lesion. Using image manipulation software (Adobe® Photoshop® CS2, Adobe Systems Incorporated; San Jose, California, USA) the lesion was gradually reduced in the sequential images to simulate repigmentation at different percentages. Participants were asked to indicate the degree of repigmentation that they considered worthwhile after 9 months of treatment, followed by the minimum level of repigmentation they would be prepared to accept.

### Online discussion groups

#### Participants

Survey participants who indicated interest in further involvement in research were invited to participate in an online discussion groups. All participants received a £10 amazon e-voucher.

Invitations were sent by email, with an information sheet attached. In total, 57 initial invitations were sent. Reminder emails and further invitations were sent if necessary, to ensure that 6–8 people were confirmed for each of the three discussion groups [9]. To aid participation, two groups were held in the evening. We used online discussion groups to make it easier for participants to join the discussion (rather than having to travel) and to make it easier for them to talk about more personal aspects of their experience with vitiligo (which might have been more difficult if they had attended in person).

To ensure familiarity with the concepts involved and the context in which the patient assessment of treatment response would be placed, confirmed participants were sent reading material on clinical research methods and primary outcome measures (see Additional file 1). Participants were also advised to read information on vitiligo from the NHS Choices web pages [10] and to watch a short video explaining clinical trials from the Medical Research Council via YouTube [11].

### Hosting the online discussion groups

Online discussion groups were used in favour of face-to-face focus group discussions in order to facilitate engagement with a broad range of participants from throughout the UK [12-14].

The online discussion groups were hosted in a private chat room based within the Vitiligo Society’s web pages. All participants followed an email link, registered for the group and, once approved by the moderator (ST), were given access to the chat room for the time of their online discussion. Prior to the groups taking place, participants were sent information about the objectives of the groups, and about consent to participate. Participants gave consent at time of registration and were encouraged to use an alias if they wished to remain anonymous.

Each group lasted for approximately 90minutes, to allow adequate time for discussion whilst avoiding participant fatigue. Participants were able to type text and send emoticons as in a standard chat room. Groups were facilitated by two to three members of the research team.

The discussion groups were semi-structured; a list of prompts was prepared in advance and these were inserted into the discussion thread at relevant time points. This ensured similarity between the questions asked of each group. Page links were created for images and inserted into the discussion thread at relevant times during the discussion to allow participants to view images of vitiligo before and after treatment and a selection of measurement scales.

Examples of prompts used in online discussion groups:

Discussion prompts were used to direct participants’ attention to relevant points. Here are examples of prompts used under various themes covered by the online discussion groups:

Prompt number and details

Theme: Most important concepts when assessing treatment success

The survey results showed that the three main areas of importance to people with regards to judging treatment success were (in order of frequency):

1. Colour match between their vitiligo and normal skin i.e. how well it blends in

2. How noticeable the vitiligo is

3. A reduction in the size of the white patches.

Which of these do you think is the most important if we are trying to capture a measure of treatment success?

Theme: Wording of questions about how noticeable vitiligo is

Let's try some example questions that ask about how noticeable your vitiligo is. These questions can be used at the end of a trial to ask people about how successful their treatment is. What do people think about these possible questions? Does the wording seem right?

Q1 How noticeable do you feel your vitiligo is, compared with the start of treatment?

Q2 How successful do you feel the treatment has been, in terms of how noticeable the vitiligo is?

Q3 How satisfied are you with how noticeable the vitiligo is?

Q4 Compared to before treatment, how noticeable is your vitiligo now?

Theme: Using the agreed question format to assess treatment response for whole body versus individual lesions

If some areas of vitiligo respond well to treatment and some do not, do you think that this question is useful to measure how noticeable the vitiligo is on all body sites affected? Or do you think that the question is only useful for assessing individual patches of vitiligo?

Adequate time was allowed after insertion of each prompt to allow participants to respond and discuss with each other freely. The facilitators guided discussion with the aim of trying to achieve consensus, and summarised the discussion findings at various intervals to check that all participants were in agreement.

At the end of the discussion group, a copy of the entire discussion thread was downloaded and saved.

### Sample size and participant selection

The sample size for this study was dictated by the time and resources available. However, we aimed to include at least 100 participants in the survey (assuming a confidence interval of 95%, and an accuracy rate or +/− 10%) and 18 – 20 participants in the discussion groups, in order to gather a broad selection of views.

For the discussion groups, purposive sampling was used to ensure diversity in terms of ethnicity and age within the groups. Using SPSS 21 Software, potential participants who had responded to the online survey were split into three groups – parents/carers of those with vitiligo (n = 13), people with vitiligo aged 17–45 (n = 45) and those aged 46 and above (n = 76). We tried to take participants’ ages into consideration when forming the discussion groups given the potentially greater familiarity with technology and “text speak” in younger participants [13]. Invitations were sent to all parents/carers, plus a random selection from other age groups. All potential participants from non-white ethnic backgrounds were invited, as well as a random selection of those from white backgrounds. This was to enable discussion of treatment for vitiligo in the context of different skin types.

### Statistical analysis

Survey results were analysed using SPSS Statistics 21 software. Results were presented descriptively. Responses to open questions were analysed thematically by a researcher (ST) and checked by a second researcher (JB) for agreement. This allowed for comparison between themes emerging from open and closed questions to be made, as well as allowing for ranking of themes by popularity overall for use in the discussion groups.

The main objective of the discussion groups was to seek consensus regarding the most appropriate wording of the question to ask people about the response of vitiligo to treatment. Whilst more formal methods of consensus development (e.g. Delphi and Nominal Group Technique) are the focus of much academic consideration, informal consensus groups such as those employed here are commonly employed in health care settings [15]. To counter some of those criticisms that informal mechanisms for consensus reaching lack ‘control’, ‘focus’ and ‘scientific credibility’ [15] here data is handled and analysed systematically following an adapted version of Template Analysis [16,17]. Template analysis utilises a hierarchical model to organise text in order to aid interpretation. In this case each point of consensus was taken as a higher-level organising code within which to summarise and consider the group discussion. So for each point of agreement the discussion which led toward this was considered and coded to reflect those factors which contributed towards the consensus and those which were a barrier. This process was completed for each discussion group with a final template constructed to include all statements where agreement spanned the different groups. This mode of analysis provides greater depth in understanding consensus, both mapping where agreement occurred and charting the process and factors which generated it. Two researchers independently checked the copy of each discussion group thread to ensure that all relevant points had been adequately identified and extracted with any discrepancies discussed with a third researcher. Consensus points are summarised below, with examples of key comments made by participants. The qualitative aspect of this study adhered to the RATS guidelines for reporting qualitative research modified for BioMed Central [18].

## Results

### Participants

**Figure 1:**
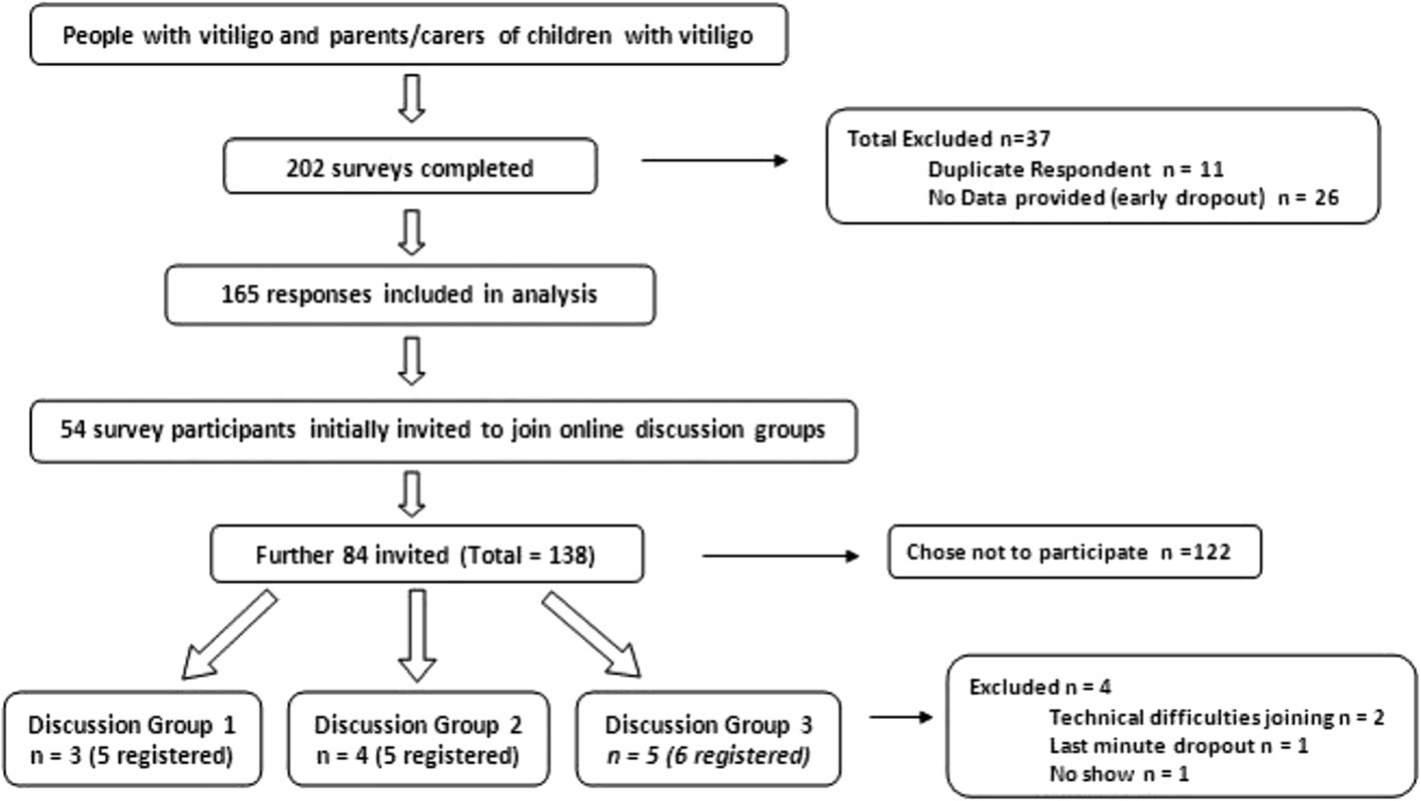
**Participant flow diagram.** Flow diagram to show participant numbers lost and included throughout the study process.

In total, 202 survey responses were received. Of these, 165 (82%) were included in analyses, and of these, 154 (76%) were fully completed surveys (Figure 1).

Responses were excluded for the following reasons:

•If the survey had been completed from the same Internet Protocol (IP) address more than once:

○ The first completely filled survey was included, and the rest excluded.

○ If multiple surveys were completed fully, only the first was included.

•The same two rules applied for duplicate email addresses given

•If the survey had not been completed past question 1, it was excluded.

The only exception to these exclusion criteria was where email addresses and demographic responses indicated that two different individuals had responded from the same IP address, so both sets of responses were included.

Baseline characteristics of the survey participants are summarised in Table 1.

**Table 1:** Demographic/other characteristics of survey respondents

**Characteristic**	**Online survey**	**Online discussion groups**
	**N = 165**	**N = 12**
*Responses completed on behalf of – n (%)*		
*Themselves*	149 (90.3)	12 (100)
*Other*	14 (8.5)	0 (0)
*Child with vitiligo*	13(92.9)	
*Spouse with vitiligo*	1 (7.1)	
*Themselves and other(s)*	2 (1.2)	0 (0)
*Unknown*	0 (0)	0 (0)
*Age – n (%)*		
<5 years	1 (0.6)	0 (0)
5-16 years	10 (6.1)	1 (8.3)
17-30 years	17 (10.3)	2 (16.7)
31-45 years	43 (26)	3 (25)
46-65 years	55 (33.3)	3 (25)
> 65years	29 (17.6)	3 (25)
Unknown	10 (6.1)	0 (0)
*Ethnicity- n (%)*		
White British	117 (70.9)	9 (75)
White Irish	1 (0.6)	0 (0)
Other White Background	15 (9.1)	0 (0)
Any Other Mixed Background	1 (0.6)	0 (0)
Indian/British Indian	9 (5.5)	1 (8.3)
Pakistani/British Pakistani	1 (0.6)	0 (0)
Bangladeshi/British Bangladeshi	2 (1.2)	1 (8.3)
Caribbean/British Caribbean	1 (0.6)	1 (8.3)
African/British African	1 (0.6)	0 (0)
Other	7 (4.2)	0 (0)
Unknown	10 (6.1)	0 (0)
*Country of residence – n (%)*		
UK	135 (81.8)	11 (91.7)
USA	12 (7.3)	1 (8.3)
Europe (excluding UK)	3 (1.8)	0 (0)
Australia	1 (0.6)	0 (0)
Asia	1 (0.6)	0 (0)
Dual – UK and Other	2 (1.2)	0 (0)
Unknown	11 (6.7)	0 (0)
*Duration of Diagnosis- n (%)*		
6-12 months	3 (1.9)	0 (0)
1-2 years	3 (1.9)	0 (0)
2-5 years	12 (7.7)	2 (16.7)
5-10 years	26 (16.8)	1 (8.3)
> 10 years	111 (71.6)	9 (75)
*Percentage of skin affected (estimate) - n (%)*		
0-10%	37 (23.3)	1 (8.3)
10-25%	49 (30.8)	4 (33.3)
25-50%	33 (20.8)	5 (41.7)
50-80%	24 (15.1)	2 (16.7)
>80%	16 (10.1)	0 (0)
Unknown	6 (3.6)	0 (0)
*Area (s) of skin affected – n (%) NB: More than one response could be given*		
Face/Neck	129 (78.2)	10 (83.3)
Body	110 (66.7)	7 (58.3)
Arms	113 (68.5)	8 (66.7)
Legs	109 (66.1)	8 (66.7)
Hands	127 (77)	10 (83.3)
Feet	113 (68.5)	9 (75)
I’d rather not say	2 (1.2)	0 (0)
Other (responses included: under arms, genitalia and hair)	35 (21.2)	2 (16.7)
*Treatments used previously – n (%) NB: More than one response could be given*		
No treatment	57 (34.5)	2 (16.7)
Topical corticosteroid	41 (24.8)	3 (25)
Protopic (Tacrolimus)	46 (27.9)	6 (50)
Elidel (Pimecrolimus)	6 (3.6)	0 (0)
Vitamin D derived cream or ointment (e.g. calcipotriol)	5 (3)	2 (16.7)
UVB	33 (20)	5 (41.7)
PUVA	20 (12.1)	2 (16.7)
Not Sure	1 (0.6)	0 (0)
Other (Responses included diet changes and alternative therapies)	28 (17)	3 (25)

The majority of participants were aged between 31 and 65 years of age and had had vitiligo for more than 10 years. One hundred and thirty three (80.6%) of those completing the survey were from white ethnic backgrounds, and 135 (81.8%) were from the UK.

### Results of survey

Question 1: “When thinking about repigmentation of vitiligo after treatment, what does a ‘cosmetically acceptable result’ mean to you?”

There were 143 responses to this open question. Multiple themes per response were allowed, yielding a total of 237 items of information coded from the 143 responses. The three most common themes related to the concept of the skin returning to normal and the vitiligo patches being less visible or noticeable. Reduction in the size of the lesion was ranked 4^th^ and was mentioned in just 12.2% of responses. The main themes to emerge are summarised in Table 2.

**Table 2:** ‘When thinking about repigmentation of vitiligo after treatment, what does a “cosmetically acceptable result” mean to you?’ (Themes in descending order of popularity)

**Theme**	**Number of responses**	**Percent**
Blends well with skin	45	19%
Less noticeable	35	14.8%
Skin back to normal	31	13.1%
Reduction in white patches	29	12.2%
Confident/Comfortable	25	10.5%
Repigment visible sites	19	8%
Any Improvement	17	7.2%
Mostly repigmented	14	5.9%
Cosmetics	9	3.8%
Unaffected by tanning	5	2.1%
Means nothing	4	1.7%
Lasting repigmentation	3	1.3%
Completely depigmented	1	0.4%

Six responses were not relevant to the question, such as “I have given up on treatment after various unsuccessful attempts”. Four respondents (1.7%) stated specifically that a ‘cosmetically acceptable result’ was not meaningful to them and not an encouraging phrase.

Question 2: “The list below gives some possible words or phrases used to describe treatment results in vitiligo. Please tell us the words/phrases that best reflect how you would judge whether or not a vitiligo treatment has worked (please tick up to THREE options)”

This question received 157 responses. The most popular words/phrases are summarised in Table 3. Eighteen responses (4.3%) were given under the category ‘Other’, and most were not relevant to treatment response, such as “Never had treatment”.

*Question 3*: *“When thinking about the repigmentation of vitiligo after treatment, do you think that ‘cosmetic acceptability of result’ and ‘satisfaction with the result’ mean the same thing?”*

**Table 3:** Popularity of Words/Phrases to describe treatment results for vitiligo

**Words/Phrases**	**Number of votes**	**Percent**
Good colour match between treated vitiligo patches and normal skin	72	17%
Skin is back to normal	66	15.6%
Feel better about appearance of skin	58	13.7%
Reduction in area of skin affected by vitiligo	48	11.3%
Even pattern of repigmentation	43	10.2%
Cosmetically acceptable result	26	6.1%
Satisfied with result	23	5.4%
Worth continuing with treatment	21	5%
Other	18	4.3%
Worthwhile result	17	4%
Result of treatment is acceptable	9	2.1%

In total, 159 responses were given for this question, with 88 (55.3%) answering ‘No’, 46 (28.9%) answering ‘Yes’ and 25 (15.7%) ‘Not sure’.

An open comment box allowed respondents to give further details, which suggested that participants felt that ‘cosmetic acceptability’ was a medical view or that of someone else and that ‘satisfaction with the result’ was a more personal and patient-led view. An example response was “The second statement suggests that the person is happy with the result whereas the first statement sounds more medical…”. In addition, negative views about the term ‘cosmetic acceptability’ were given, such as “rather vague”, “impersonal” and “implies vitiligo only affects skin”.

Question 4: “Please give us any other suggestions on the questions we should ask people about the result of vitiligo treatment.”

The main theme that emerged was asking questions regarding psychological factors, (36 responses; 34.6%) such as individual feelings, confidence, and comfort in wearing fewer clothes. The next emerging themes were details about the treatment (19 responses; 18.3%) and the duration of the improvement (14 responses; 13.5%). Other results are summarised in Table 4.

Question 5: “After 9 months of vitiligo treatment, which of the pictures below shows a level of treatment response that you feel would be worthwhile to you? Please choose the letter associated with the chosen image.”

**Table 4:** Themes emerging from “other suggested questions”

**Theme**	**Number of Responses**	**Percent**
Psychological	36	34.6%
Treatment details	19	18.3%
Improvement duration	14	13.5%
Was the treatment worth the results	8	7.7%
Adverse effects	6	5.8%
Satisfaction	6	5.8%
Back to normal	4	3.8%
Has colour returned	3	2.9%
Reduction in white patches	3	2.9%
Sun protection	2	1.9%
Would they do it again	2	1.9%
Make it simple	1	1%

The 158 responses are summarised in Table 5. Almost 80% of participants said that they would consider at least an 80% improvement to be a worthwhile result after 9 months of treatment (equating to images G H and I).

*Question 6:* “*After 9 months of vitiligo treatment, which of the pictures below represents the MINIMUM treatment response that you would be prepared to accept?”*

**Table 5:** Worthwhile treatment response and minimum level of response acceptable for after 9 months

**Treatment response image (Approximate% repigmentation)**	**Worthwhile treatment response**	**Minimum treatment response acceptable**
	**Number of votes (cumulative %)**	**Number of votes (cumulative %)**
A (20)	2 (1.3)	8 (5.1)
B (30)	0 (1.3)	8 (10.2)
C (40)	3 (3.2)	7 (14.7)
D (50)	7 (7.6)	17 (25.6)
E (60)	8 (12.1)	16 (35.9)
F (70)	12 (19.8)	26 (52.6)
G (80)	18 (31.6)	43 (80.2)
H (95)	57 (77.7)	22 (94.3)
I (100)	51(100)	9 (100)

The 156 responses are summarised in Table 5. Similar to Question 5, when asked to identify the MINIMUM acceptable treatment response, the results were heavily skewed towards high levels of repigmentation. Sixty four percent of participants wanted to see at least a 70% improvement after 9 months of treatment (images F, G, H and I), although improvement of as little as 50% was also considered worthwhile for some.

### Key messages to be explored in discussion groups

The survey revealed some key messages, which were taken forward to the online discussion groups for further exploration.

The first of these was that there were three main areas of importance to people when judging treatment success, namely: colour match between their vitiligo and normal skin, i.e. how well it blends in; how noticeable the vitiligo is; and a reduction in the size of the white patches.

Another key message for further exploration was that the majority of respondents said they would consider 80% or more repigmentation to be worthwhile treatment response after 9months and that the minimum they would be prepared to accept would be 70% repigmentation.

### Results of discussion groups

Three online discussion groups were held, involving a total of 12 participants (n = 4; n = 3 and n = 5 respectively). Participants ranged in age from 16 to over 65 years old, and had been affected by vitiligo for between four and 27 years. An additional four participants were registered to join the groups, but did not participate because they had technical difficulties in accessing the chat room, or were unavailable at the last minute. Due to participant availability, we were not able to run separate discussion groups for participants of different age groups, as we had planned. However, this did not seem to have an adverse effect on any participants’ ability to contribute to discussions. A total of 50 pages (approximately 16,000 words) of text were obtained from the three groups and analysed as described above.

### Summary of areas of consensus

All three groups achieved consensus both within and between the individual discussion groups in several areas.

Points for which there was consensus across the groups included:

i. The most important concept when asking about success of treatment response is “how noticeable the vitiligo is after treatment”.

ii. A scale with five response options (both words and numbers) is the best scale to use when answering the question

Question: Compared to before treatment, how noticeable is the vitiligo now?

a. More noticeable (1)

b. As noticeable (2)

c. Slightly less noticeable (3)

d. A lot less noticeable (4)

e. No longer noticeable (5)

iii. A score of 4 or 5 on the above five-point scale would represent a successful treatment response

iv. The question should only be used to assess individual vitiligo lesions, rather than all areas affected by vitiligo

These areas of consensus are discussed in more detail below.

### Most important concept: How noticeable vitiligo is after treatment

In response to the question regarding the most important concept when judging treatment success, all three groups were unanimous that the most important concept was how ‘noticeable’ the vitiligo is after treatment. Although some participants initially felt that other concepts were important, after further discussion with other participants, consensus was soon reached, with minimal input from the facilitators. Moreover, several participants commented that the ‘noticeability’ of the vitiligo was a useful ‘catch-all’ phrase which covered elements of the other two concepts (colour match/blending and a decrease in size of the lesions). For example:

‘Of the three you have written, I think 1 and 3 are covered by 2’

‘I would say, most noticeable first, as 1 and 3 determine this’

Participants in all groups acknowledged that for people with paler skin tones, ‘noticeability’ may be less of an issue than for people with darker skin:

Participant: *I am lucky in that I have very fair skin so my condition is not that easy to notice……… but I imagine it is a big issue if it can be seen.*

Facilitator: *Do you think that if your vitiligo was in visible areas, that how noticeable it was would be the most important to you?*

Participant: *Yes*

Having established that the ‘noticeability’ of the vitiligo was the most important concept, participants then decided on the best wording of the question to ask trial participants in order to establish the ‘noticeability’. Prompts used for this discussion are shown in the ‘examples of prompts used in online discussion groups**’** subsection.

There was agreement that asking about “satisfaction” alongside the notion of “noticeability” was confusing for patients, as the two terms have contradictory implications.

‘Because noticeable to me, denotes it is noticeable-which is a negative and yet I am being asked how satisfied I am-which is a bit confusing’

There was rapid development of consensus in the first two groups that Q4 (‘Compared to before treatment, how noticeable is your vitiligo now?’) was the most appropriate and easy to understand, and the third group agreed with this:

‘Love it- uncomplicated and to the point’

### What scale to use when answering the question

When groups were asked about the best scale with which to measure responses to the question, a preference was expressed for a linear scale, as opposed to a scale made up of images such as pictograms or emoticons. However, participants felt that the linear scale needed to contain a reasonable number of choices:

‘I think it’s sometimes more difficult to make a judgement when there are fewer parameters’

Although the first group expressed a provisional preference for a linear scale with 10 divisions, time was limited for discussion of this point and so the discussion was developed further in groups 2 and 3. The subsequent groups felt that a 5-point scale with adjectival markers was best.

Having agreed on the question ‘Compared to before treatment, how noticeable is your vitiligo now?’ in group 1, further discussion with the participants in groups 2 and 3 showed support for the response options shown in the left-hand column of Table 6. The final wording of two responses was amended slightly after the discussion groups had been completed (so that all responses consistently included the word ‘noticeable’). The amended wording, shown in the right-hand column of Table 6, was circulated amongst all participants and there was unanimous support for it.

**Table 6:** Wording of response options

**Wording agreed during discussion groups**	**Amended wording, approved after discussion group by participants**
Worse than before (1)	More noticeable (1)
About the same (2)	As noticeable (2)
Slightly less noticeable (3)	Slightly less noticeable (3)
A lot less noticeable (4)	A lot less noticeable (4)
Hardly noticeable (5)	No longer noticeable (5)

### What score on the scale constitutes treatment ‘success?’

The groups were unanimous that a ‘successful’ treatment would need to score at least a ‘4’ on this scale (a lot less noticeable [4] or no longer noticeable [5]).

**Figure 2:**
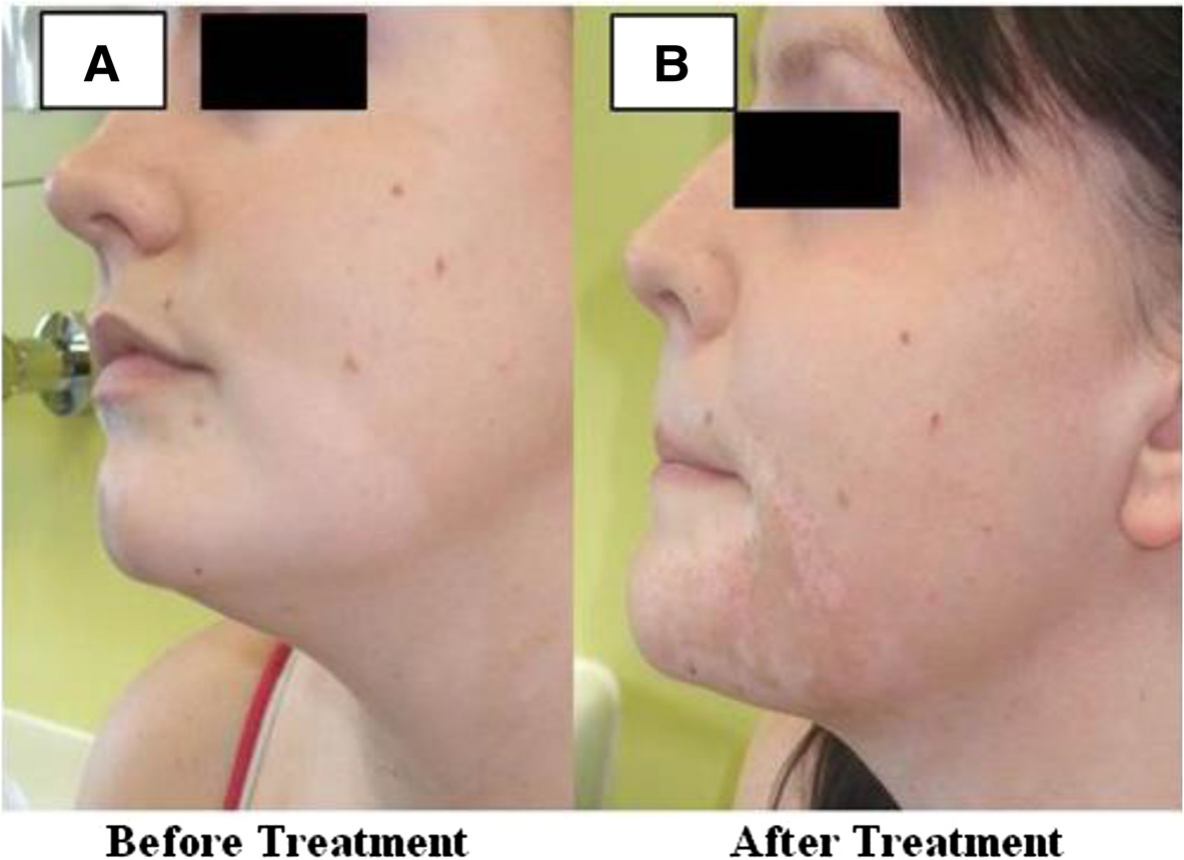
**A set of ‘before’ and ‘after’ images showing hyperpigmentation used in the online discussion groups.** A set of ‘before’ and ‘after’ images showing hyperpigmentation used in the online discussion groups. This set of images consent was gained from the individual seen in Figure 2 for use of their images in research publications as well as was obtained from images held at the Centre of Evidence Based Dermatology of before and after treatment. Full future studies.

In particular, participants expressed a preference for having both numbers and words on the scale, and for the option of ticking a box to give an answer.Participants in all groups agreed that the question and scale were suitable when assessing vitiligo lesions that have partially repigmented but which, due to hyperpigmentation or uneven repigmentation, are actually more noticeable after treatment. Here is a summary of a discussion when participants in one group were shown some ‘before and after’ images that included hyperpigmentation (Figure 2 for images):

Participant 1: *That is interesting. There is a reduction in vitiligo area but the patchiness makes it look more obvious. In spite of the partial repigmentation, I would answer 1.*

Participant 2: *On balance I think I would say 2, because the area near to the eye has responded well but the chin seems more noticeable now that it is not such a large block*

Participant 3: *To me it is more blotchy so 1*

Facilitator: *So if this was your vitiligo, would you say that treatment was successful or unsuccessful*?

Participant 2: *Unsuccessful I think*

Participant 1: *Partly successful, but if I had to opt for successful or unsuccessful, I would go for unsuccessful as it has made the vitiligo more obvious.*

Participant 3: *Unsuccessful.*

### Use of the question to refer to individual treated vitiligo lesions or all affected areas of vitiligo

In the final discussion group, participants were asked to comment specifically on whether the question could be used to ask about all areas affected by vitiligo or whether it was best used to assess specific patches (see ‘examples of prompts used in online discussion groups**’** subsection for prompts). Participants were unanimous that the question should be specific to target areas, and that this was particularly important for visible sites.

### Use of the question with children and their parents/carers

The third group was also asked about the suitability of the question if the trial participant was a child, and a proxy response from a parent or carer was required:

‘I feel that parents could answer for their child as they would be fully aware of the child’s feelings’

‘I think the questions would be suitable for children, although their responses might be a bit more optimistic than adults’

‘For a child, I think noticeability will be determined by their peers - a parent may or may not have good insight into this’

## Discussion

### Summary of main findings

This work has provided valuable insight into how patients with vitiligo evaluate treatment success and has laid the foundation for creating a validated patient-rated outcome measure for use in future trials of vitiligo treatments.

Although the concept of a ‘cosmetically acceptable result’ had previously been identified as an important measure of treatment success amongst people with vitiligo [2], our initial survey work showed that this term was rather unhelpful to patients, who felt that it was vague, impersonal and rather ‘medical’; or it implied that vitiligo was just something to be covered up (using cosmetic camouflage).

There was good agreement between the open and closed responses to survey questions. Common themes including ‘blends well with skin’, ‘less noticeable’ and ‘reduction in white patches’, were mentioned most frequently in response to an open question. While the most popular phrases in response to a more closed question were: ‘good colour match’, ‘reduction in area of skin affected’ and ‘even pattern of repigmentation’.

‘Feel better about appearance of skin’ was another popular theme, and although this is a highly important concept, we did not pursue it further in the discussion groups because psychological response to treatment was beyond the remit of this study. Specific validated scales to assess the impact of vitiligo on psychological wellbeing have been described elsewhere [19,20] as have quality of life scales regarding physical appearance and cosmetic products [21].

Another main theme to emerge from the survey results was that many respondents equated a cosmetically acceptable result with ‘skin is back to normal’. Although this is of course the ideal result for people with vitiligo, the likelihood of vitiliginous skin fully returning to normal after treatment is low. In addition, responses to a question about the skin being ‘back to normal’ would be in a binary ‘Yes/No’ form and would not allow for a scale of more gradual increments, which is more likely to be useful when measuring the partial repigmentation expected after treatment. ‘Skin back to normal’ would also be covered by the top rating on any scale used to judge treatment success. For these reasons, we decided not to pursue this theme further during the discussion groups, focusing instead on the other three key themes that had emerged (colour match between their vitiligo and normal skin; how noticeable the vitiligo is; and a reduction in the size of the white patches).

Responses to survey questions about the minimum level of repigmentation considered to be worthwhile after a 9-month period of treatment showed that people with vitiligo generally hope for very high degrees of repigmentation; nearly 80% of respondents said they would consider 80% or more repigmentation to be worthwhile and 64% said that the minimum they would be prepared to accept would be 70% or more repigmentation. This was helpful in guiding our understanding of what patients might consider to be a clinically meaningful treatment response and corresponds with the quartile of >75% repigmentation being taken to represent treatment success in many previous vitiligo trials [2].

The online discussion groups used in the second stage of this work were very successful and popular amongst the participants. Due to the widespread availability of internet access and increasing familiarity with online means of communication, online discussion groups are emerging as a useful medium for conducting health research [12,13,22]. The study also reflects an increasing use of electronic communication to support group decision making and consensus making [22].

It was striking that the members of the online discussion groups quickly reached consensus in a number of areas. The first area of consensus was that the ‘noticeability’ of vitiligo was the most important concept when assessing treatment success, and that the ‘noticeability’ of vitiligo is a useful ‘catch-all’ concept that reflects the other two main concepts to emerge from the survey (colour match/blending and a decrease in size of the lesions).

Consensus was also reached rapidly regarding the use of a 5-point scale of responses, including words and numbers, when answering the question: ‘Compared to before treatment, how noticeable is the vitiligo now?’ Participants were happy that this scale was suitable for assessing lesions with different percentages and patterns of repigmentation, and there was strong consensus that a score of 4 or 5 on the scale equated with treatment success. Participants also agreed unanimously that if individual vitiligo lesions are treated, the noticeability of the lesions should be assessed individually, as opposed to assessing noticeability of vitiligo as a global measure for all affected areas of skin.

## Conclusions

### Strengths and limitations of the study

Limitations of this research work include the fact that the participants involved in the survey and discussion groups were almost entirely based in the UK. It is possible that the views of people with vitiligo in other countries may be quite different from those in the UK, and this may limit the external validity of the patient-rated measure. Another limitation was the absence of parents/carers of children with vitiligo from the discussion groups. We tried hard to recruit such individuals to the groups, by offering to host the groups at times that would be convenient for them, but none of the parents/carers who had participated in the survey were willing, or able, to join the discussion groups. We did, however, obtain positive feedback from parents/carers about the outcome measure after the discussion groups had taken place. Because it is not possible to know the characteristics of those who chose not to respond to the survey, it is possible that a degree of bias may have arisen due to a particular cohort of participants choosing to respond.

Given the potentially disproportionate impact of vitiligo on people with darker skin types, it was possibly disappointing that only 4.2% of study participants were from black and ethnic groups. However, this figure is representative of the mix of ethnicities in the UK population. For example, 85.8% of survey participants were white, and the Office of National Statistics estimates that 87.9% of the population are white; 7.7% of participants were Indian, Pakistani or other of Asian ethnicity, compared to the ONS estimate of 5.8% of the UK population [7].

In this work, we did not explore participants’ views on the impact of vitiligo on their quality of life. There is already and extensive literature on this subject, and there are validated vitiligo-specific quality of life scales available for assessing this [19]. Once fully validated, the outcome measure we are developing can be used in parallel with vitiligo-specific quality of life scales, to assess both the visual and psychological aspects of treatment ‘success’ from the patient’s perspective.

Qualitative research such as this can be prone to biases if carried out by one particular group (e.g. clinicians). Our group included two clinicians, a psychology student, a non-clinical Professor of applied dermatology research, and two qualitative researchers. We believe this is a suitable mix to avoid some of the potential for bias in the design and interpretation of the study.

### Implications for research

This work has demonstrated for the first time that percentage repigmentation may not be the best measure of vitiligo treatment success from a patient’s perspective. Instead, how noticeable the vitiligo patches are is a key concept for patients. Greater awareness of patients’ perspectives in judging treatment response in future clinical trials is essential. Additional work is now required to validate this measure further; in particular with respect to construct validity. We will ensure that these findings are incorporated into future international discussions regarding the most appropriate core outcome measure for inclusion in future vitiligo trials.

## Abbreviations

UVB: Ultraviolet B; PUVA: Psoralen plus ultraviolet A.

## Competing interests

The authors declare that they have no competing interests and are not in personal receipt of any funding for this or any other work.

## Authors’ contributions

SKT, JMB and KST made substantial contributions to the acquisition of data, analysis and interpretation of the data as well as making major contributions to the draft manuscript. DMW made substantial contributions to the study design of the online discussion groups and PL contributed to the analysis and interpretation of qualitative data analysis. ASWY contributed to the creation of repigmentation images to be used in online survey work. All authors read, redrafted and approved the final manuscript.

## Authors’ information

SKT BSc (Hons). Research Associate, Centre of Evidence-based Dermatology, University of Nottingham. KST BSc, PhD. Professor of Applied Dermatology Research, Centre of Evidence-based Dermatology, University of Nottingham. DMW BSc (Hons), MA, MSc, PhD. Lecturer, School of Medicine, Research Design Service, University of Nottingham. PL PhD. Lecturer, School of Medicine, University of Nottingham. ASWY BSc (Hons), MB BS, MRCP. Specialty Registrar in Dermatology, Norfolk and Norwich University Hospitals NHS Foundation Trust, Norwich, UK. JMB B MedSci, BM BS, FRCP. Consultant Dermatologist and Honorary Consultant Lecturer, Centre of Evidence-based Dermatology, University of Nottingham.

## Pre-publication history

The pre-publication history for this paper can be accessed here:

http://www.biomedcentral.com/1471-5945/14/10/prepub

## Supplementary Material

Additional file 1 Background reading material on clinical research methods and primary outcome measures given to participants before participation in online discussion groups. Click here for file
