# Supplementary material for: Survey and online discussion groups to develop a patient-rated outcome measure on acceptability of treatment response in vitiligo
Source: BMC Dermatol. 2014 Jun 14;14:10. doi: 10.1186/1471-5945-14-10 (PMC4075774; doi:10.1186/1471-5945-14-10)
Supplement: Additional file 1 — Background reading material on clinical research methods and primary outcome measures given to participants before participation in online discussion groups. [file 1471-5945-14-10-S1.pdf]

Additional file 1: Background reading material on clinical research methods and primary outcome measures given to participants before participation in online discussion groups.

Overview of clinical research methods

How does clinical research help patients and the NHS?

Clinical research can provide insights into the following areas:

- **Diagnosis** – how are diseases and conditions diagnosed? Do they run in families?
- **Cause** – what causes disease? What are the links between lifestyle and other conditions/diseases (known as co –morbidity)
- **Care** – how should disease best be treated/cared for? How can we best organise care and support? What helps patients respond to care and self care?
- **Cure** – can we cure the disease? Can we stop it coming back?

What is clinical research?

Clinical research is a branch of medical science that determines the safety and effectiveness of medications, devices, diagnostic products, and treatments intended for human use. These may be used for prevention, treatment, diagnosis or for relief of symptoms in a disease

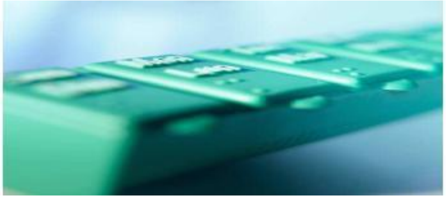

What are clinical trials?

Clinical trials evaluate the effectiveness and safety of medications or medical devices by monitoring their effects on large groups of people. They are generally developed along the following lines:

Phase 1 trials - these trials investigate the therapy in a few research subjects, who are healthy volunteers. These trials assess safety, tolerability, and how the drug works in humans. These trials are not carried out in healthcare settings, but tightly controlled clinics with 24-hour medical attention

Phase 2 trials – these increase our understanding of the study drug's safety and effectiveness in a controlled setting to decide on doses. Generally you need more than 100 patients to demonstrate relevant results, and define an appropriate patient population to do further studies in.

Phase 3 trials – only done once the treatment is deemed potentially safe and effective. These are much bigger studies involving 1000 + research subjects, usually undertaken in health care settings such as clinics/hospitals. These trials are more ‘real life’, and need to represent normal populations

to be useful in practice. Usually a comparison is made with standard treatment or a placebo. It is imperative that the drug is shown to be effective and safe in this phase.

What is a Randomised Controlled Trial?

A trial in which people are randomly allocated to receive or not receive (control group) an intervention/treatment and then followed to determine the effect of the intervention. The two or more groups should have similar types and numbers of people in them. Having a control group allows researchers to assess if the results at the end of the trial would have happened by chance, or if the treatment/intervention was having an effect.

Randomised Controlled Trial

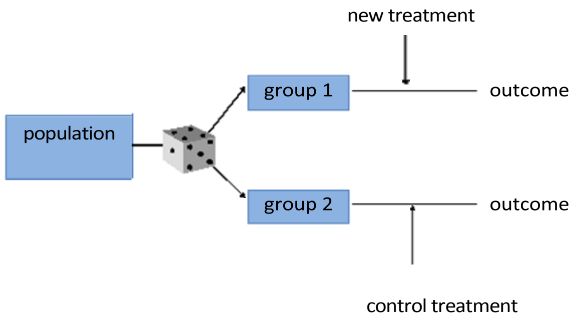

Research outcomes

These can be things that we may measure, such as thickness of skin, spreading of rash, blood pressure. Or they can be assessments of feelings and mood using questionnaires for example or they can be statistics such as death, birth, time taken to recover from operations for example.

Ideally research outcomes need to work for researchers and study participants, but also need to be recognisably important from patient/carer and clinical viewpoints too.

What is a 'primary outcome measure' in a clinical trial?

An outcome measure is a planned measurement used in trials to decide how well a treatment has worked. The measurement is carried out in all patients taking part in the trial and then the results are compared to see whether one group has done better than the other. For example, the results of the group receiving a new treatment will be compared with the results from patients receiving inactive 'placebo'.

Trials usually measure several different outcomes to look at different aspects of the condition. But the ***primary*** outcome measure is the **most important measure** in the trial. It gives the overall message of whether or not the new treatment is any better than what it is being compared to.
